# Detrimental effect of an early exposure to antibiotics on the outcomes of immunotherapy in a multi-tumor cohort of patients

**DOI:** 10.1093/oncolo/oyae284

**Published:** 2024-10-19

**Authors:** Patricia Guerrero, Víctor Albarrán, Carlos González-Merino, Coral García de Quevedo, Pilar Sotoca, Jesús Chamorro, Diana Isabel Rosero, Ana Barrill, Víctor Alía, Juan Carlos Calvo, Jaime Moreno, Patricia Pérez de Aguado, Pablo Álvarez-Ballesteros, María San Román, Juan José Serrano, Ainara Soria, María Eugenia Olmedo, Cristina Saavedra, Alfonso Cortés, Ana Gómez, Yolanda Lage, Álvaro Ruiz, María Reyes Ferreiro, Federico Longo, Eva Guerra, Íñigo Martínez-Delfrade, Pilar Garrido, Pablo Gajate

**Affiliations:** Department of Medical Oncology, Ramón y Cajal University Hospital, 28034 Madrid, Spain; Department of Medical Oncology, Ramón y Cajal University Hospital, 28034 Madrid, Spain; Department of Medical Oncology, Ramón y Cajal University Hospital, 28034 Madrid, Spain; Department of Medical Oncology, Ramón y Cajal University Hospital, 28034 Madrid, Spain; Department of Medical Oncology, Ramón y Cajal University Hospital, 28034 Madrid, Spain; Department of Medical Oncology, Ramón y Cajal University Hospital, 28034 Madrid, Spain; Department of Medical Oncology, Ramón y Cajal University Hospital, 28034 Madrid, Spain; Department of Medical Oncology, Ramón y Cajal University Hospital, 28034 Madrid, Spain; Department of Medical Oncology, Ramón y Cajal University Hospital, 28034 Madrid, Spain; Department of Medical Oncology, Ramón y Cajal University Hospital, 28034 Madrid, Spain; Department of Medical Oncology, Ramón y Cajal University Hospital, 28034 Madrid, Spain; Department of Medical Oncology, Ramón y Cajal University Hospital, 28034 Madrid, Spain; Department of Medical Oncology, Ramón y Cajal University Hospital, 28034 Madrid, Spain; Department of Medical Oncology, Ramón y Cajal University Hospital, 28034 Madrid, Spain; Department of Medical Oncology, Ramón y Cajal University Hospital, 28034 Madrid, Spain; Department of Medical Oncology, Ramón y Cajal University Hospital, 28034 Madrid, Spain; Department of Medical Oncology, Ramón y Cajal University Hospital, 28034 Madrid, Spain; Department of Medical Oncology, Ramón y Cajal University Hospital, 28034 Madrid, Spain; Department of Medical Oncology, Ramón y Cajal University Hospital, 28034 Madrid, Spain; Department of Medical Oncology, Ramón y Cajal University Hospital, 28034 Madrid, Spain; Department of Medical Oncology, Ramón y Cajal University Hospital, 28034 Madrid, Spain; Department of Medical Oncology, Ramón y Cajal University Hospital, 28034 Madrid, Spain; Department of Medical Oncology, Ramón y Cajal University Hospital, 28034 Madrid, Spain; Department of Medical Oncology, Ramón y Cajal University Hospital, 28034 Madrid, Spain; Department of Medical Oncology, Ramón y Cajal University Hospital, 28034 Madrid, Spain; Department of Medical Oncology, Ramón y Cajal University Hospital, 28034 Madrid, Spain; Department of Medical Oncology, Ramón y Cajal University Hospital, 28034 Madrid, Spain; Department of Medical Oncology, Ramón y Cajal University Hospital, 28034 Madrid, Spain

**Keywords:** antibiotics, immune checkpoint inhibitors, microbiota, solid tumors

## Abstract

**Background:**

Immune checkpoint inhibitors (ICI) have changed the therapeutic landscape of many solid tumors. Modulation of the intestinal microbiota by antibiotics (Abx) has been suggested to impact on ICI outcomes.

**Methods:**

Retrospective analysis of 475 patients with advanced solid tumors treated with ICI from 2015 to 2022. For each patient, the use of Abx was recorded from 1 month before ICI initiation until disease progression or death. The impact of Abx on objective response rates (ORR), disease control rates (DCR), progression-free survival (PFS), and overall survival (OS) was analyzed. Kaplan-Meier and log-rank tests were used to compare survival outcomes.

**Results:**

In total 475 patients with advanced solid tumors were evaluated. Median age was 67.5 years and performance status (PS) was 0-1 in 84.6%. 66.5% of patients received Abx during treatment with ICI, mainly beta-lactams (53.8%) and quinolones (35.9%). The early exposure to Abx (from 60 days before to 42 days after the first cycle of ICI) was associated with a lower ORR (27.4% vs 39.4%; *P* < .01), a lower DCR (37.3% vs 57.4%; *P* < .001), lower PFS (16.8 m vs 27.8 m; HR 0.66; *P* < .001]) and lower OS (2.5 m vs 6.6 m; HR 0.68; *P* = .001]). The negative impact of Abx on OS and PFS was confirmed by a multivariable analysis. This effect was not observed among patients receiving Abx after 6 weeks from ICI initiation.

**Conclusions:**

Our results validate the hypothesis of a detrimental effect of an early exposure to Abxon the efficacy of ICI in a multi-tumor cohort of patients.

## Teaser

The use of antibiotics modifies intestinal microbiota, potentially affecting treatment outcomes. This real-world study evaluated the effect of antibiotic exposure within different periods of time on the clinical outcomes of a multi-tumor cohort of patients treated with immune checkpoint inhibitors.

**Implications for practice**

Early exposure to antibiotics in solid tumor patients undergoing immunotherapy modifies the microbiota, potentially impacting treatment outcomes adversely. Clinicians should judiciously consider antibiotic use, as preserving microbiota diversity may enhance immunotherapy efficacy.

## Background

Immune checkpoint inhibitors (ICI) have become an essential part of the therapeutic arsenal against most solid tumors. Their widespread use has prompted the search of predictive biomarkers of efficacy, and understanding their potential interactions with other drugs has become essential to optimize their use in clinical practice. Previous studies have suggested that exposure to antibiotics (Abx) may have an influence on response rates among patients treated with ICI due to the subsequent modulation of intestinal microbiota.

The microbiota is a complex ecosystem encoded by around 3 million genes, comprising the microbiome.^1^ It serves as an intermediary between the host and the external environment^2^ and plays multiple functions, including anti-inflammatory and antioxidant effects, and homeostasis regulation.^3,4^

In recent decades, extensive research has been conducted concerning the potential impact of intestinal microbiota on the etiopathogenesis of various diseases. Intestinal conditions such as inflammatory bowel disease or colon cancer can lead to alterations in the microbiota. On the other hand, the incidence of several conditions seems to be increased by alterations in the microbiota.^5^

Furthermore, it has been observed that the microbiota may both undergo detrimental changes induced by some commonly used drugs, and also modulate host sensitivity to their pharmacological effects. The main hypothesis underlying this phenomenon is the ability of intestinal bacteria to transform the composition of drugs and alter their bioavailability.^2^ Stimulation of the cellular immune response through various bacterial products has also been described,^6^ suggesting that changes in the microbiota could potentially impact on the individual responsiveness to IT.

Based on the premise that Abx may modify the intestinal microbiota, we have performed a real-world study evaluating the impact of Abx exposure within different periods of time on the clinical outcomes of a multi-tumor cohort of patients treated with ICI.

## Methods

### Study design and population

We have conducted a retrospective study at our institution (Ramón y Cajal University Hospital, Madrid), with prior approval from the Clinical Investigation Ethical Committee (CEIC).

We included patients with unresectable or metastatic tumors who received ICI between April 2015 and October 2022. All patients were adults (>18 years) and had provided informed consent in writing to receive the treatment. Patient data were extracted from pharmacy databases and electronic institutional medical files. We excluded those who received combinations of ICI and chemotherapy or any other anticancer agents, as well as those who received ICI in the context of clinical trials.

For each patient, we collected demographic data, relevant medical history (autoimmune diseases, previous history of transplantation, or human immunodeficiency virus [HIV] infection) and performance status (PS) according to the Eastern Cooperative Oncology Group (ECOG) score.^7^ It was recorded in the electronic medical records of all patients before starting the first cycle of ICI (C1). Regarding oncological history, we collected information about the diagnosis date, tumor histology, cancer stage according to the American Joint Committee on Cancer [AJCC] staging system, and disease burden. Previous use of other anticancer therapies and type of ICI, together with their respective toxicities, were also recorded. Clinical radiology reads were utilized to assess response, with radiologists utilizing RECIST v. 1.1 methods as standard practice in routine clinical reads. Between 2 and 5 target lesions were chosen, and their largest diameter was measured in pretreatment scans as a baseline. Radiologists later used their baseline measurements to compare the response in subsequent images. In cases where these standardized measurements were not available, the authors recalculated them ourselves. We considered the date of progression the date of the image scan where progressive disease was reported.

Patients were divided into those who did not receive Abx and those who did. The latter group was further divided into 3 subgroups: those who received Abx from 60 days before the start of the C1 of ICI to 42 days afterward (group 1: −60D, +42D, also referred to as those receiving early abx), those who received Abx from 42 days after de C1 of ICI to up to 6 months afterward (groups 2: +42D, +6m), and those who received Abx beyond the first 6 months after the start of ICI (groups 3: >6m). The route of administration and the duration of the treatment received were not recorded, although it was considered that an antibiotic cycle corresponded to a period of 7-14 days. In addition, the different antibiotic families used were documented, including beta-lactams (penicillins, cephalosporins, carbapenems, and monobactams), quinolones, macrolides, aminoglycosides, lipopeptides, glycopeptides, dalbavancin, linezolid or tedizolid, metronidazole, clindamycin, tetracyclines, fosfomycin, nitrofurantoin, rifamycins, and cotrimoxazole.

### Statistical analysis

We used STATA software to perform statistical analysis. All statistical evaluations were 2 sided. The distribution of qualitative variables was summarized using percentages and frequencies. To evaluate the association between them, we used Fisher’s exact or chi-square tests. The distribution of continuous variables was summarized using the mean and standard deviation. Kaplan-Meier curves were used for the estimation of survival outcomes. The log-rank test was used for multivariable logistic regression analysis. Statistical significance was set at *P* values < .05.

## Results

### General characteristics

A total of 475 patients with metastatic or unresectable solid tumors were included, the majority of whom were male (66.74%), with a mean age of 66.74 years (Table 1). Thirty four patients (7.16%) with a previous diagnosis of autoimmune disease and 13 (2.74%) with controlled HIV infection were included. Regarding PS before the first cycle of immunotherapy, 147 patients (31.01%) had PS = 0, 254 (53.59%) had PS = 1, and 73 (15.4%) had PS ≥ 2. PS prior to C1 was higher in those patients that were on antibiotic treatment around ICI initiation (PS 0: 38.2% vs 22.3%).

**Table 1.** Baseline characteristics of the patients.

Clinical characteristics		Total (*n:* 475)	No Abs (*n*: 259)	Abs (−60D, + 42D) (*n*: 216)	*P* value
Sex	Male	317 (66.7%)	173 (66.8%)	144 (66.7%)	
	Female	158 (33.3%)	86 (30.9%)	81 (37.5%)	.97
Type of tumor	NSCLC	161 (33.9%)	81 (31.3%)	82 (37.9%)	
	Urothelial	82 (17.2%)	43 (16.6%)	39 (18.1%)	
	Renal	65 (13.7%)	39 (15.1%)	26 (12%)	
	Melanoma	53 (11.2%)	33 (12.7%)	20 (9.3%)	.97
	Head/neck	52 (10.9%)	24 (9.3%)	28 (13%)	
	Others	62 (13.1%)	39 (15.1%)	21 (9.7%)	
Type of ICI	Anti-PD1	306 (64.4%)	171 (65.9%)	135 (63.2%)	
	Anti-PDL1	97 (20.4%)	47 (18.2%)	50 (23.2%)	
	Anti-PD1 + anti-CTLA4	63 (13.3%)	36 (13.9%)	27 (12.5%)	.90
	Anti-CTLA4	9 (1.9%)	5 (1.9%)	4 (1.8%)	
Line of treatment	1^st^ line	200 (42.1%)	105 (40.5%)	95 (44%)	
	2^nd^ line	205 (43.2%)	112 (43.2%)	93 (43%)	.55
	≥ 3^rd^ line	70 (14.7%)	42 (16.3%)	28 (13%)	
ECOG prior to ICI	0	147 (31.0%)	99 (38.2%)	48 (22.3%)	
	1	254 (53.6%)	133 (51.4%)	121 (56.3%)	<.01
	≥ 2	73 (15.4%)	27 (10.4%)	46 (21.4%)	
Disease burden	Brain	74 (15.6%)	35 (13.5%)	39 (18.1%)	.16
	Liver	120 (25.3%)	59 (22.8%)	61 (28.4%)	.16
	Lung	216 (45.6%)	122 (47.1%)	94 (43.7%)	.46
	Bone	133 (28.1%)	68 (26.3%)	65 (30.2%)	.34

The most represented tumor in our sample was NSCLC with 161 cases (33.89%), followed by urothelial cancer (17.26%), renal cancer (13.68%), melanoma (11.16%), and head and neck squamous tumors (10.95%). Less represented tumor subtypes included gastrointestinal tumors (4.42%), cervical/endometrial cancer (1.89%), non-melanoma skin cancer (1.68%), malignant mesothelioma (1.05%), salivary gland tumors (0.63%), breast cancer (0.42%), sarcoma (0.42%), tumors of unknown origin (0.42%), tumors of vagina/vulva (0.42%) and 6 others (1.26%) (Table 1).

Most patients received IT as the first (42.11%) or second-line (43.16%) treatment. 161 patients received pembrolizumab (33.89%), 136 received nivolumab (28.63%), 81 received atezolizumab (17.05%), 63 received nivolumab plus ipilimumab (13.26%), 14 received avelumab (2.95%), and 20 received other ICI (4.21%) (Table 1).

More than half of the patients (66.5%) received Abx during treatment with immunotherapy, and 45.5% did it around ICI initiation (from −60D to D42). The most frequently used families were beta-lactams (53.8%), quinolones (35.9%), carbapenems (10.9%), and macrolides (10.9%). It is noteworthy that almost a quarter of our sample received 3 or more cycles of antibiotic therapy during the above-mentioned period (11.0%: 3 cycles and 13.8% ≥4 cycles), with the duration of a cycle being between 7 and 14 days. One quarter of the patients 25.7% received 1 cycle and 16.0% received 2 cycles (Table 2).

**Table 2.** Exposure to antibiotics.

Exposure to Abs	Any time	316 (66.5%)
	−60D to D42	216 (45.5%)
	D42 to 6m	151 (31.8%)
	>6 m	94 (19.8%)
N of cycles of Abs	1 cycle	122 (25.7%)
	2 cycles	76 (16%)
	3 cycles	52 (11%)
	≥ 4 cycles	66 (13.9%)
Type of Abs	Penicilines	213 (57.3%)
	Quinolones	174 (46.8%)
	Carbapenems	76 (20.5%)
	Cephalosporins	79 (21.3%)
	Macrolides	49 (13.2%)
	Glycopeptides	43 (11.6%)

### Influence of antibiotics on clinical outcomes

**Figure 1.**
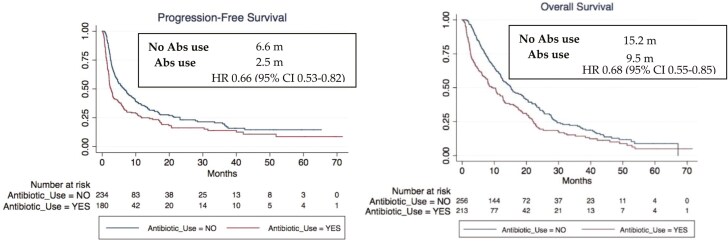
Impact of Abs exposure on ICI clinical outcomes.

Early exposure to Abx (-60D-D42) had a clinically significant impact on the four clinical parameters we analyzed versus those who did not receive Abx: In this subgroup, a decrease in the overall response rate (ORR) was observed (27.4% vs 39.4%; *P* < 0.01), as well as a lower DCR (37.3% vs 57.4%; *P* < 0.001), lower PFS (2.5 m vs 6.6 m; HR 0.66 [95% CI, 0.53-0.82; *P* < 0.001]) and lower OS (9.5 m vs 15.2 m; HR 0.68 [95% CI, 0.55-0.85; *P* = .001]). This was confirmed by a multivariate study including PS, tumor type, disease burden, type of ICI, line of treatment and antibiotic treatment at another time (*P* = .01). This effect was not significant for patients receiving Abx in D42-6m (ORR: 46% vs 39%, *P* 0.2; PFS: 6.5 m vs 5.8 m, *P* 0.25; OS: 15.6 m vs 15.2 m, *P* 0.87) and >6m vs those who did not receive Abx (PFS: 23.1 m vs 17.6 m, *P* 0.09; OS: 52.3 m vs 32.6 m, *P* 0.08; Figure 1).

We also conducted an analysis of the most commonly used antibiotic families in our study. We found that penicillins (ORR 26.8% vs 27.8%, *P* 0.88) and quinolones (ORR 29.9% vs 24.2%, *P* 0.35) do not influence the response to IT. We did not perform this analysis on other antibiotic families because there was not a sufficient sample size to ensure representative results and also, because most patients received more than one different antibiotic, potentially confounding the data.

## Discussion

The negative impact of the exposure to Abx on the efficacy of IT has already been suggested in previous studies. The first retrospective study that suggested an influence of Abx on ICI clinical outcomes^8^ analyzed 249 patients with NSCLC, renal cell carcinoma, and urothelial carcinoma exposed to Abx within 2 months before and 1 month after the initiation of IT, showing a statistically significant decrease in OS. Subsequently, similar results have been found by several other studies,^9-11^ reporting worse PFS and OS among patients exposed to Abx.

Other parameters, such as the type of antibiotic used, the timing of prescription, or the route of administration, have been evaluated by previous studies. In a cohort of 60 patients with metastatic solid tumors,^12^ the impact of antibiotic exposure within 2 weeks before or after the first cycle of immunotherapy was analyzed. A statistically significant decrease in the response rate (RR) (25% vs 61%) (*P* = .02) was observed only in the subgroup receiving broad-spectrum antibiotics (defined as those that cover Gram-positive and negative ± anaerobic bacteria), while this effect was not observed among those receiving narrow-spectrum antibiotics (only covering Gram-positive bacteria). Other studies have evaluated the potential influence of the route of administration of the antibiotic and the type of infection, showing worse outcomes when the antimicrobial was administered intravenously or when dealing with lower respiratory or genitourinary tract infections.^13^ The relevance of the Abx cycle duration has also been assessed. Galli et al^14^ found that Abx administration over a short period did not seem to impact on the prognosis compared to longer cycles. PFS and OS were significantly lower in patients with a longer than average exposure to Abx (defined as the percentage of antibiotic treatment days between days of immunotherapy): 2.2 vs 7.7 months, *P* < .0001; 4.9 vs 16.3 months, *P* = .0004, respectively. While these are interesting hypotheses, they may be biased by other factors, such as the spectrum of antibiotics administered, as suggested in the aforementioned study.^12^ In general, intravenous administration and longer antibiotic cycles are associated with the use of broad-spectrum antibiotics for the management of severe infections in admitted patients. The impact on clinical outcomes based on the family of Abx has also been analyzed. In a study by Lu et al^15^ of 127 patients receiving broad-spectrum Abx, the use of fluoroquinolones was correlated with poorer results in terms of OS (121 days vs 370 days, *P* = .047). In the analysis by antibiotic families that we conducted in our study, we did not find differences in outcomes based on the different families used. However, given that this is a retrospective study with a small sample size, we cannot draw firm conclusions.

In accordance with our results, previous studies have shown an impact on clinical outcomes only for the early administration of Abx around treatment initiation. In a meta-analysis^16^ of 23 studies including NSCLC patients receiving ICI, a 6-month decrease in mOS was observed among those exposed to Abx from −60D to +60D; a slightly wider time window than the used in our study (−60D, +42). Similarly, late exposure to Abx regarding the initiation of IT also does not show an impact on clinical parameters (as mentioned in our results, there were no differences in terms of PFS, OS, DCR, and ORR in patients receiving Abx beyond 42 days after the first cycle). This highlights the importance of the timing of antibiotic therapy administration, as it appears to be detrimental just around the initiation of treatment with checkpoint inhibitors.

Even though we’re still unsure of the reason behind this temporal association, the impact of other factors like corticosteroid exposure is also more significant on clinical outcomes when it happens early on.

To the best of our knowledge, this is the first study evaluating the late exposure to antibiotics in long responding patients, since more of the previous works have assessed their effect around treatment initiation.

It could be argued that patients exposed to Abx tend to have more clinical comorbidities and/or disease-related complications, which may intrinsically lead to worse prognosis. However, our multivariable analysis suggests that the early exposure to Abx has a negative impact on response rates and survival outcomes irrespective of potential confounding factors such as PS or disease burden. In addition, previous data reported in the literature^17^ have also suggested a negative influence of early Abx exposure on IT response, independent of performance status or tumor extent.

Due to the retrospective nature of our study, it may be subject to various limitations, primarily due to the small sample size, which results in some subgroups being underrepresented. Additionally, there are other drugs frequently used in oncology patients that can also influence the microbiota and were not considered when analyzing the data (such as proton pump inhibitors, corticosteroids, antidepressants, or aspirin). Other data regarding the antibiotic itself, such as the route of administration used or the indication, could provide useful information for our study. Another limitation to mention is the use of day +42 as the cutoff point for the data collection. Although we chose it based on previous data described in the literature, it is not possible to determine the exact point at which antibiotic exposure ceases to be detrimental.

The mechanism underlying the potential impact of Abx on ICI clinical outcomes remains to be properly understood. The most plausible hypothesis for this effect is the negative modulation exerted by antibiotics on the intestinal microbiota. Raymond et al^18^ demonstrated how the administration of cefprozil for 7 days led to a statistically significant loss of the metagenome from intestinal bacteria. Other studies have observed a higher diversity of bacterial subspecies in the fecal material from patients who have not received Abx compared to those who have.^19,20^

Thanks to Next-Generation Sequencing (NGS) techniques (such as shotgun metagenomics) and novel cultivation methods,^21^ bacterial strains that lead to a favorable or detrimental modulation of the host’s immune response have been identified.

In mouse models with similar phenotypic characteristics, the administration of antiPD1, antiPDL1, or antiCTLA4 antibodies seems to lead to worse antitumor activity in the absence of certain immunogenic intestinal bacteria, such as *Akkermansia muciniphila, Bacteroides fragilis, Bifidobacterium spp., Collinsella aerofaciens,* and *Faecalibacterium spp*.^19,22^

Frankel et al^23^ conducted the first prospective study demonstrating differences in the microbiota composition between responders and non-responders to IT. Fecal material from 39 subjects with metastatic melanoma treated with ICI was analyzed. It was observed that certain types of intestinal bacteria were enriched in all responding patients, regardless of the type of ICI received (*Bacteroides caccae*), while others were increased according to the type of antitumor treatment administered, such as *Bacteroides thetaiotamicron* in responders to ipilimumab plus nivolumab or *Dorea formicogenerans* in responders to pembrolizumab. In a study performed by Routy and colleagues,^19^ fecal material from patients with NSCLC and clear cell renal cancer who had received IT was analyzed through shotgun sequencing. It was observed that certain bacterial populations were enriched in responders to IT and were absent in non-responders. Recently, a significant suppressive role of gram-negative bacilli in non-responding patients has been suggested.^24^ Some other studies^22,25^ have observed differences in the composition of intestinal microbiota between responders and non-responders to IT, supporting the relevance of the microbiota in the modulation of antitumor immune response.

The pathophysiological mechanism through which intestinal bacteria can modulate the immune response is not entirely clear. Recent studies have defined the microbiota as the “immune-cancer set point.”^6,26^ An increase of interferon-gamma-producing CD8+ T lymphocytes in the colon, and the enhanced activation of dendritic cells with high expression of class I major histocompatibility complex (MHC) molecules have been described as potential mechanisms. In patients with pancreatic ductal adenocarcinoma undergoing surgery with long-term survival, a more diverse microbiota (including *Streptomyces, Pseudoxanthomonas,* and *Saccharopolyspora*) was observed,^27^ which induced CD8+ T cell-dependent immune responses. The modulation of tumor-infiltrating CD4+ T lymphocytes and changes in the ratio of regulatory T lymphocytes to CD8+ T lymphocytes,^28^ may also be relevant for immune modulation. In preclinical models, these findings correlated with an increased efficacy of anti-PD-1 antibodies,^29,30^ proving how the composition of the microbiota may impact on antitumor immune response.

Despite the evidence regarding the negative impact of Abx use on the intestinal microbiota, thus potentially interfering with responsiveness to IT, addressing this issue in clinical practice has proven to be challenging. It has been observed that even when shorter courses of Abx are administered, microbiota reconstitution after Abx discontinuation is suboptimal, especially among patients with a baseline less favorable microbiota.^18^ Additionally, avoiding Abx exposure is not always possible in patients with advanced tumors, due to the high incidence of disease and treatment-related infectious complications.

The use of fecal microbiota transplantation (FMT) to favorably modulate gut microbiota is a novel strategy currently being evaluated by several studies. In mouse models, it has been described that the oral intake of *Bifidobacterium* improves tumor response and has synergistic effects when administered concomitantly with anti-PDL-1 antibodies.^31^

Nowadays, the abundance of bacterial families correlated with a better response to immunotherapy in patients’ fecal material can be used as a biomarker to predict which patients will be responders. There are studies which have aimed to replicate the microbiota of responders to IT by transplanting their feces into recipient patients. Using high-throughput sequencing techniques, the degree of engraftment of the donor’s microbiota into the recipient’s can be analyzed. Want et al.^32^ showed that changes in bacterial families that comprise the patient’s microbiota can be observed some days after FMT. This technique has been proven effective in patient series with other conditions such as immune-related colitis, refractory *Clostridium difficile* infection, or inflammatory bowel disease.^33–35^

Although occasional severe adverse effects have been reported, such as sepsis or fatal intestinal mucormycosis,^36–38^ treatment with FMT or probiotic intake has been shown to be generally safe.^39^ Further research is required to verify the safety and clinical efficacy of that FMT to improve clinical outcomes of IT.

## Conclusions

Our study is one of the largest real-world multi-tumor cohorts that evaluate the impact of Abx in IT efficacy. Our results support the hypothesis of a detrimental effect of early exposure to antibiotics on the efficacy of immune checkpoint inhibitors, as we have observed a negative impact on clinical responses and survival outcomes. This fact has already been suggested in previous studies, although in smaller cohorts that included fewer types of tumors than in our study. A better understanding of the role that our microbiota plays in the modulation of antitumor immune response is necessary for the development of novel therapeutic strategies, such as FMT, to improve the results of immunotherapy in poor responders.

## Data Availability

The data underlying this article will be shared on reasonable request to the corresponding author.
